# Effect of apple vinegar on folliculogenesis and ovarian kisspeptin in a high-fat diet-induced nonalcoholic fatty liver disease in rat

**DOI:** 10.1186/s12902-022-01205-1

**Published:** 2022-12-23

**Authors:** Fahimeh Shams, Monireh Aghajani-nasab, Mahsa Ramezanpour, Razieh Habibipour Fatideh, Fahimeh Mohammadghasemi

**Affiliations:** 1 Cellular and Molecular Research Center, Department Of Anatomy, Guilan University Of Medical Sciences, Rasht, Iran; 2 Cellular and Molecular Research Center, Department of Biochemistry and Biophysics, School of Medicine, Guilan University of Medical Sciences, Rasht, Iran; 3 Medical Biotechnology Research Center, School of Paramedicine, Guilan University of Medical Sciences, Rasht, Iran; 4 Department of Biochemistry and Biophysics, School of Medicine, Guilan University of Medical Sciences, Rasht, Iran; 5 Cellular and Molecular Research Center, School of Medicine, Guilan University of Medical Sciences, P.O.BOX: 3363, Guilan Rasht, Iran

**Keywords:** Obesity, High fat diet, Sex hormones, Vinegar, Kisspeptin, Ovary

## Abstract

**Background:**

Non-alcoholic fatty liver disease (NAFLD) adversely affects reproduction. We aimed to study the effect of a high-fat diet (HFD), supplemented with apple vinegar, on folliculogenesis in a rat model of NAFLD.

**Methods:**

Female rats were randomly divided into four groups (*N* = 28): Standard diet (SD), SD + vinegar, HFD, and HFD + vinegar groups. At the end of the study, biochemical tests were assessed in serum. HOMA-IR (Homeostatic model assessment-Insulin resistance) was calculated. Sex hormones were determined using an ELISA kit; ovary follicle counts were studied using histological methods. The proliferation index of granulosa cells was determined using immunohistochemistry. Kisspeptin expression in the ovary was detected using RT-PCR.

**Results:**

The HFD induced steatohepatitis and NAFLD**.** The ovaries in the rat model of NAFLD were atrophied. The ovaries had less count of developing follicles and corpus luteum, and more degenerated and cystic follicles in comparison with the SD group**.** Vinegar + HFD consumption decreased ALT, compared to the HFD group (*P* = 0.004). Steatohepatitis was reduced in the Vinegar + HFD group (*P* = 0.001). Vinegar + HFD considerably reduced HOMA-IR (*p* = 0.01). The HFD + vinegar diet could increase estradiol (*P* = 0.001), without significantly affecting progesterone or testosterone. In addition, an increase of primordial follicles as an ovarian reserve and also primary follicles were determined in the HFD + vinegar group. There were no statistical differences in the granulosa cell proliferation index in various follicle types between groups. HFD + vinegar significantly enhanced ovarian kisspeptin expression (*p* = 0.04).

**Conclusions:**

The vinegar diet in a rat model of NAFLD raises estradiol, primordial, and small primary follicles, and increases ovarian kisspeptin expression indirectly. Insulin resistance and obesity were improved by apple vinegar, and anti-glycemic and anti-lipidemic effects were also determined. The supplementation of apple vinegar in NAFLD might be useful for ovary. However, it requires further investigation.

## Background

In non-alcoholic fatty liver disease (NAFLD), significant fat deposition in the liver can occur even if the patient does not drink large amounts of alcohol. It has a strong connection to diabetes and excessive weight gain [1]. The clinical significance of NAFLD is due to its high incidence (20 − 30%), in the general population and its potential progression to end-stage liver disease and, in rare cases, hepatocellular carcinoma [2]. Given that the frequency of NAFLD is significantly higher in obesity, type 2 diabetes mellitus, and dyslipidemia, the significance of insulin resistance (IR) in the development of this condition has been examined and a strong correlation has been demonstrated between the two entities [2].

In humans, the concept of a high-fat diet (HFD) ranges from 30 to 75 percent of calorie intake [3]. Even in rats, HFD intake has been shown to cause reproductive dysfunction, reduced ovarian reserve, altered ovarian gene expression, increased ovarian inflammation, and aberrant oocyte development [4, 5]. A high-fat, high-sugar diet affects the ovarian shape, causes polycystic ovaries, decreases corpus luteum development, and increases ovarian androgen production [6].

Kisspeptin is a new hypothalamic peptide that promotes the release of endogenous gonadotrophin-releasing hormone (GnRH). Kisspeptin boosts circulation luteinizing hormone (LH) levels in women following a single subcutaneous bolus dose [7]. In 2006 for the first time, Castellano et al. demonstrated that the genes encoding kisspeptin and also its receptors are expressed in the adult rat ovary during the different phases of the estrous cycle [8].

Kisspeptin–KISS1R signaling has a critical function in ovarian physiology and kisspeptin is a crucial regulator of oocyte maturation and maintenance of the oocyte pool [7].

Kisspeptin treatment increases oocyte maturation in subfertile women undergoing in vitro fertilization (IVF) therapy [9]. Kisspeptin may also be involved in the regulation of ovarian steroidogenesis, folliculogenesis, and ovulation [9]. It has been shown that HFD decreases kisspeptin expression in rat ovarian tissue [10].

Several studies have demonstrated that vinegar and organic acids have therapeutic benefits in treating insulin resistance [11, 12], obesity [13], and hyperlipidemia. Ingestion of vinegar improves insulin sensitivity in type 2 diabetes [11, 14]. It has long been recognized that vinegar offers health benefits due to its antioxidant, anti-obesity, antidiabetic, anticancer, antibacterial, and hypolipidemic properties [15].

Apple vinegar also improves spermatogenesis testicular function by reducing HOMA-IR, Lee index, apoptosis of germ cells, and increasing total antioxidants in a rat model of NAFLD [16]. Little is known about NAFLD and female reproductive function. NAFLD has recently been hypothesized to be a clinical syndrome that affects body organs. It likely affects the ovaries, becoming a determining factor for poor folliculogenesis. We hypothesized that vinegar by ameliorating the symptoms of metabolic syndrome may indirectly recover the folliculogenesis and function of the ovary in a rat model of NAFLD. Therefore in this study, we assessed biochemical tests, insulin resistance, ovary function, expression of ovarian kisspeptin, and histological features of the ovary.

## Methods

### Rat model study

The animals were manipulated according to the ethical principles and guidelines for conducting Medical Research in Iran. All the procedures for studies on animals were approved by the Ethics Committee of Gulan University of Medical Sciences, (Approval, December 01, 2018). (ethical code: IR.GUMS.REC.1397.307). All applicable international, national, and/or institutional guidelines for the care and use of animals were followed. All methods are reported in accordance with ARRIVE guidelines.

Animals were randomly separated into four groups after one week of acclimatization (*N* = 28). Group 1, the standard diet (SD) group (*n* = 7) received an SD containing 10% kcal fat [17].

Group 2, the SD + vinegar group (*n *= 7) received an SD containing 10% kcal fat for the first 16 weeks, and then they received a combination of SD and apple vinegar (5 g vinegar powder/100 g SD) for further 8 weeks (total 24 weeks).

Group 3, the HFD or NAFLD group (*n* = 7) was fed a diet containing 60% kcal energy.

for 24 weeks. HFD was prepared using lard. This animal has been demonstrated to be prone to developing steatohepatitis (NAFLD) over 24 weeks [17].

Group 4, the HFD + vinegar group (*n* = 7) received 60% kcal fat for the first 16 weeks, and then, they received a combination of HFD and vinegar (5 g vinegar powder/100 g HFD) for further 8 weeks (total 24 weeks).

The powder of apple vinegar contained 5 g/dL of acetic acid and was then, pelleted into diets [18]. The Isfahan Royan Institute for biotechnology generated all diet formulas and energy estimations for diets (Table 1). All animals were provided with food and drink on an ad libitum basis. All animals were chosen during their estrous cycle. At the end of the experiment, each group.

**Table 1:** Animal groups and their diets

**Groups**	**Number/group (n)**	**Diet**
**SD: standard diet**	*n* = 7	Standard diet containing 10%kcal fat for 24 weeks
**SD + V: standard diet + vinegar**	*n* = 7	Standard diet for 16 weeks and apple vinegar diet (5 g vinegar powder/100 g standard diet) for further 8 weeks (total 24 weeks)
**HFD: high fat diet**	*n* = 7	High fat diet containing 60% kcal energy from lard for 24 weeks
**HFD + vinegar**	*n* = 7	HFD for 16 weeks and apple vinegar (5 g vinegar powder/100 g HFD) for further 8 weeks (total 24 weeks)

### Animal surgery

After week 24, estrous-phase animals were weighed and their naso-anal length was measured to determine the Lee index.

After the trials, all animals were anesthetized with an intraperitoneal (i.p.) simultaneous injection of 87 mg ketamine/kg of body weight and 13 mg of xylazine/kg [19]. Biochemical testing was performed on blood drawn from the inferior vena cava. Both ovaries were surgically removed. Liver tissue also was sampled for histological studies.

For fixation and histological examinations, the left ovary and liver samples were immersed in 10% neutral buffered formalin. The right ovary was used for real-time polymerase chain reaction (RT-PCR) and molecular studies. Histological examinations of liver tissue were also performed.

### Lee index

Lee index was calculated as follows: [Lee index = (weight/naso-anal length) × 1000].

Over 310, animals were classified as obese.

### Determination of the estrous cycle

Vaginal cytology was used to study the estrous cycles of all animals. On a slide, vaginal smears were created. Then, they were stained with Papanicolaou (PAP) stain after drying. The estrous cycle is divided into four phases: proestrus, estrous, metestrus, and diestrus. Proestrus is characterized by a high ratio of nucleated to cornified epithelial cells. During the estrous stage, a substantial proportion of cornified eosinophilic cells is also present. The metestrus phase is characterized by a high concentration of leukocytes and a sparse population of cornified epithelial cells. Diestrus is characterized by the presence of major leukocytes [20].

### Biochemical assays

Blood was centrifuged for ten minutes. Separated serum was stored at -80 °C until the biochemical experiment was completed. Biochemical studies were performed to determine the blood glucose, serum cholesterol, triglyceride (TG), and High-density lipoprotein (HDL) levels using a diagnostic kit (Pars Azmoon, Iran). The insulin levels were measured using an ELISA kit from Demedite, Germany. The kit had a specificity of 0.1 ng/mL. Pars Azmoon photometric diagnostics kits were used to detect aspartate aminotransferase (AST) and alanine aminotransferase (ALT). The kits have a specificity of 2 and 4 U/L, respectively. The Pars Azmoon photometric kit was used to determine the activity of all enzymes.

HOMA-IR (Homeostatic Model Assessment-Insulin Resistance) was calculated as follows: HOMA-IR = Insulin (mU/L) × glucose (mmol/L)/22.5

### Measurement of total antioxidant capacity (TAC)

TAC levels in serum were measured using a TAC test kit from Zell Bio GmbH (Germany).

The sensitivity of the kit was 0.1 mM. The total antioxidant capacity was determined according to the instructions included in the kit.

### Analysis of sex hormones

Blood concentrations of estradiol and progesterone were determined using an ELISA kit (Monobind, USA) with a sensitivity of 8.2 pg/mL and 0.1 ng/mL, respectively. The testosterone concentrations were determined using an ELISA kit (Abnova, USA). The testosterone test has a sensitivity of 0.5 µg/mL and a specificity of 0.1 µg/mL. All hormone testing procedures were performed exactly as specified in the kit's instructions.

### Liver histopathology

Histology, and thus a liver biopsy, is considered the gold standard for confirming NAFLD [21]. The liver specimens were fixed in 10% neutral buffered formalin, dehydrated, and embedded in paraffin for histological examinations. Then, three slices were cut from each specimen, and at least five fields in each slide were processed for histological assessment. All tissues were examined using a light microscope. Steatosis was considered normal when the total steatosis in three sections within each field was less than 5%; mild steatosis occurred when the overall steatosis was between 5 and 33%. Steatosis was considered moderate if it was in the range of 33% to 66%. Finally, if it exceeded 66%, it was called severe [16]. Inflammatory cells were counted in representative lobules and expressed as a percentage for histological investigation of the liver. Inflammation was quantified at a 200 × magnification in three zones of the liver's characteristic lobules: zone I (port area), zone II (middle area), and zone III (central vein area) [16].

### Ovary histopathology and follicle count

For routine tissue preparation, paraffin-embedded fixed ovarian tissues were used.

The tissues were then sectioned at a thickness of five microns and stained with hematoxylin and eosin (H&E), using a microtome (Leitz, Germany). Follicle counts were performed on every fifth ovary slice for histological examination, with each section separated by approximately ten from the subsequent section utilized for quantification [22]. The sections were examined using an Olympus light microscope.

The number of primordial, primary, pre-antral, antral, corpus luteum, atretic, and cystic follicles was determined. Follicles were divided morphologically. The smallest follicles were primordial follicles. They contained a single oocyte and a single layer of follicular squamous cells. Primary follicles were larger than primordial follicles, containing an oocyte and a single layer of cuboid follicular cells. Pre-antral follicles lack an antral cavity and have two or more layers of granulosa cells. Antral or Graafian follicles have one or more small antral cavities (early antral) or one large antral cavity (late antral); both were included in the current study.

Atretic follicles were determined by at least two criteria, including the presence of granulosa cells with a pyknotic nucleus, separation of the granulosa layer from the basal membrane, damaged zona pellucida, and oocyte segmentation. Cystic follicles were identified by their huge antral cavity filled with fluid, the absence of an oocyte, a thin granulosa layer, and a thick theca layer. Corpora lutea were also counted. To overcome double counting, we numbered only follicles having oocyte-carrying nuclei [20].

### Proliferative index of Ki-67 immunohistochemistry

The proliferation index of granulosa cells was determined using immunohistochemistry and the Ki-67 marker. We employed a rabbit monoclonal antibody directed against the mouse Ki-67 (Zytomed, Germany). Staining was carried out as reported earlier. The proliferation indices of granulosa cells were calculated by multiplying the total number of cells by the number of Ki-67 positive cells. After multiplying by 100, the result was expressed as a percentage [20].

### Detection of kisspeptin expression in the ovary using RT-PCR

The components of the ovarian tissue were immediately frozen in liquid nitrogen, pending further investigation. Total RNA was extracted according to the manufacturer's instructions using the Sinaclon kit (Iran). After isolating the RNA, it was treated with DNase to eliminate any possible contamination.

The cDNA was synthesized using a cDNA synthesis kit (BioFACT™, South Korea).

The relative mRNA expression of kisspeptin was determined by RT-PCR, using ABI equipment (StepOne™, USA). To normalize gene expression, the glyceraldehyde 3-phosphate dehydrogenase (GAPDH) gene was employed as a control. Primer3web was used to generate polymerase chain reaction (PCR) primers for the aforementioned genes (version 4.0.0).

Primer-BLAST, which is available at the National Center for Biotechnology Information (NCBI), was used to validate the created PCR primers. The primer sequences for PCR are summarized in Table 2.

**Table 2:** Primer sequences used for quantitative real-time PCR

**Gene symbol**	**Forward sequence 5'-3'**	**Reverse sequence 5'-3'**	**Annealing temperature (°C)**
Kisspeptin	TGCTGCTTCTCCTCTGTGTCGC	CAGGCTTGCTCTCTGCATACCGC	58
GAPDH (Ref)	GAACATCATCCCTGCATCCA	CCAGTGAGCTTCCCGTTCA	58

### Statistical analysis

Statistical analysis was performed using SPSS software version 26 and Graph Pad Prism version 7. The Kolmogorov–Smirnov (KS) test was used to determine the normality of the data distribution. The continuous data were reported as Mean ± Standard Error. To compare data among groups, the analysis of variance (ANOVA) test was used, followed by the post hoc Tukey test. When the data were not normally distributed or the homogeneity of variance assumptions was violated, the Kruskal–Wallis non-parametric test was applied. *P *< 0.05 was chosen as the degree of significance.

## Results

### Vinegar diet attenuated HFD-induced alteration in lipid profile in the rats with NAFLD

Serum cholesterol, TG, and HDL levels were determined in all groups as the primary lipid profile markers. Our findings indicated that rats with HFD-induced NAFLD had significantly higher serum TG (*p* = 0.004) and lower HDL(*p* = 0.001) concentrations, compared to the SD group. Vinegar + HFD consumption significantly decreased cholesterol and TG, and increased HDL, compared to the HFD group (*P* = 0.01) (Table 3).

**Table 3:** Effect of vinegar on biochemical tests in NAFLD rats

**Parameter**	**SD**	**HFD**	**SD + V**	**HFD + V**
**Lee Index**	293.14 ± 11.04	329.64 ± 21.734^a**^	288.80 ± 8.855^b**^	304.67 ± 6.586^b**^
**Cholesterol(mg/dl)**	72.142 ± 10.457	112.777 ± 3.209^a**^	66.125 ± 7.458^b**^	81 ± 4.546^b*^
**Triglyceride(mg/dl)**	104.285 ± 19.238	179 ± 9.296^a*^	85.250 ± 8.612^b**^	107.181 ± 14.837^b**^
**HDL (mg/dl)**	64 ± 1.632	44.111 ± 1.274^a**^	64.25 ± 3.288^b**^	61 ± 3.144^b**^
**ALT (U/L)**	59.571 ± 8.989	91.666 ± 6.071^a*^	55.25 ± 4.738^b**^	47.4 ± 3.646^b**^
**AST (U/L)**	69.857 ± 9.723	139 ± 11.061^a**^	65.5 ± 5.168^b**^	121.9 ± 8.496^a≠^
**TAS (mM)**	2.926 ± 0.15	1.364 ± 0.205^*^	2.354 ± 0.252	2.14 ± 0.198

Data are expressed as Mean ± SE. There are 7 rats in each group (*n* = 7). a: Significant data in comparison to the SD, b: Significant data compared to the HFD group

^*^P = 0.004

^**^P = 0.001

≠ *P* = 0.002

### Vinegar diet reduced Lee index in the rats with NAFLD

To assess the HFD diet's effect on excessive weight gain, all animals were weighed and the Lee index was calculated. After 24 weeks on the HFD, animals were substantially more obese than the SD group (*p* < 0.05). However, HFD + apple vinegar had a significant effect on the Lee index, compared to HFD animals (*p* = 0.01) (Table 3).

### Vinegar diet reversed HFD-induced insulin resistance in the rats with NAFLD

**Fig. 1:**
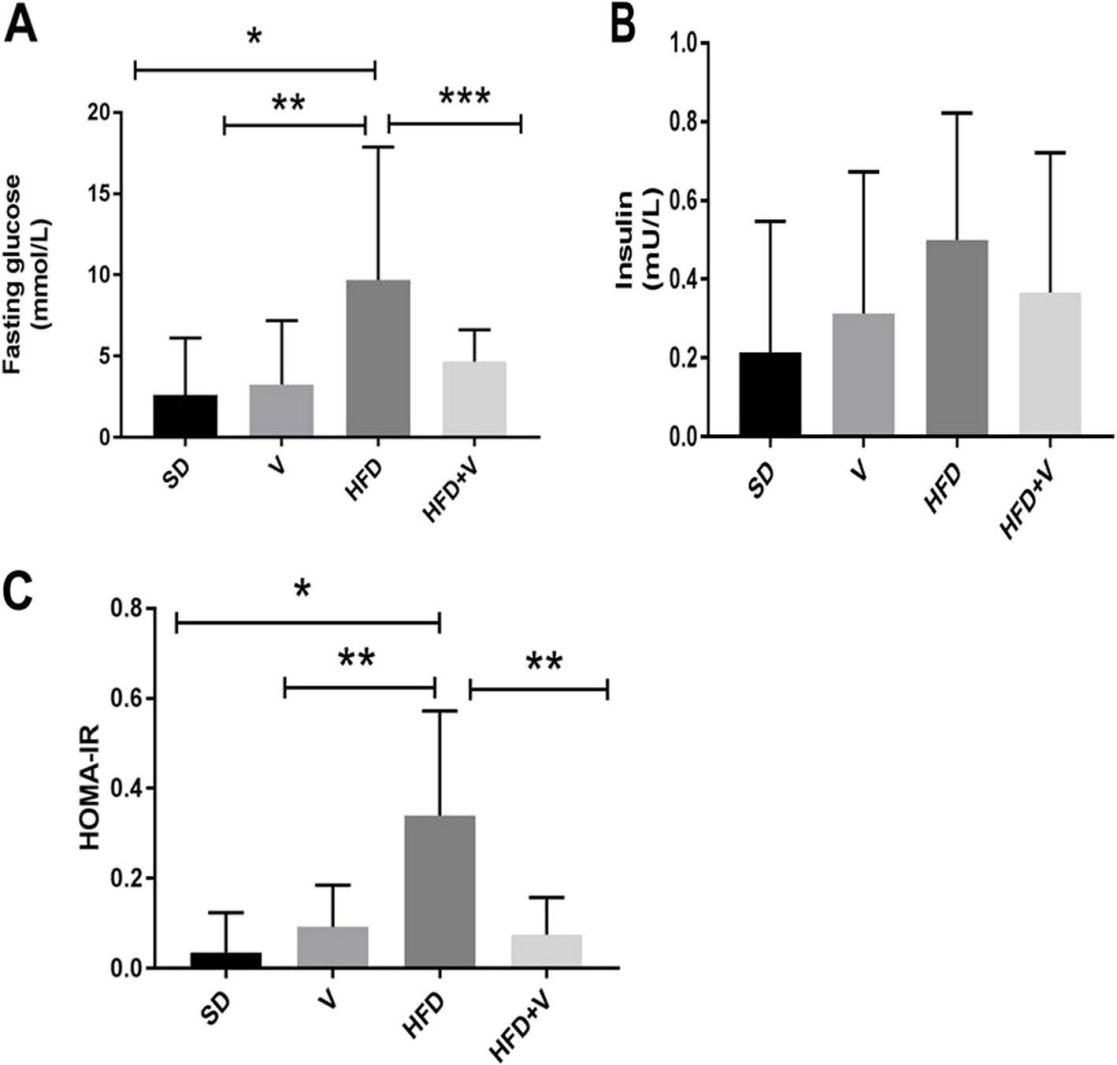
Serum concentration of glucose, insulin and calculated HOMA-IR in rats in different groups. Data are expressed as Mean ± SE. There are 7 rats in each group (*n* = 7). **A**: shows fasting glucose, *p = 0.004, **p = 0.01 and ***p < 0.05. **B**: shows serum insulin and **C**: shows HOMA-IR, *p = 0.001 and **p = 0.01

Given that hyperinsulinemia is usually included as a measure of metabolic dysfunction in rats with NAFLD, all animals had their insulin resistance tested. Blood samples were used to determine fasting insulin and glucose levels, and HOMA-IR was also determined. Fasting glucose levels were considerably higher in the HFD group than in the SD group (*p* = 0.004). However, their insulin levels were not significant. Additionally, HFD animals demonstrated insulin resistance, as evidenced by their higher HOMA-IR scores, compared to the SD group (*p* = 0.001) and vinegar group (*p* = 0.01), (Fig. 1). Vinegar + HFD significantly decreased HOMA-IR (*p* = 0.01) and glucose levels (*p* < 0.05), compared to the HFD group (Fig. 1).

### Vinegar diet improved liver enzymes in the rats with NAFLD

In comparison with the SD group, a high-fat diet significantly elevated serum levels of ALT (*p* = 0.004) and AST (*p* = 0.001). While HFD + vinegar consumption prevented ALT from growing significantly, in comparison to HFD alone (*p* = 0.001). In comparison to the HFD, the HFD + vinegar group had a non-significant drop in AST levels. (Table 3).

### Vinegar diet attenuated HFD-induced alteration in liver histology in the rats with NAFLD

**Fig. 2:**
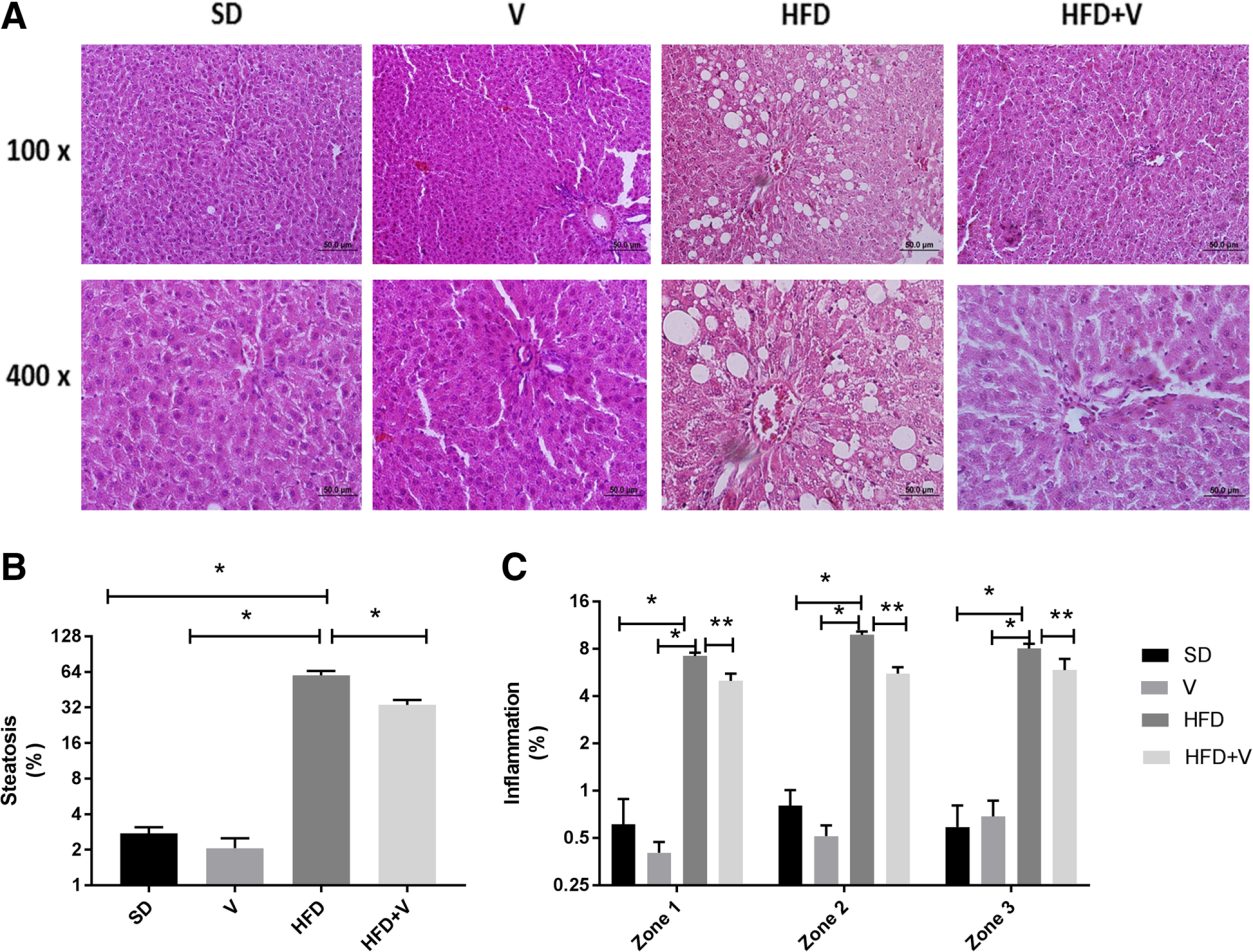
Photomicrograph of rat liver. Each column is showing a separate group. Data are expressed as Mean ± SE. There are 7 rats in each group (*n* = 7). **B**: shows steatosis % in hepatocytes, *p = 0.001, **D**: shows inflammation in three zones of hepatic lobules, *p = 0.001 and **p < 0.01

The HFD produced macro and micro steatosis at a rate of approximately 60% (moderate steatosis), compared to less than 5% in the SD group ( *p* = 0.001). When compared to the HFD group, HFD + vinegar reduced steatosis to (32.65%) mild steatosis (*p* = 0.001) (Fig. 2).

Additionally, the HFD caused inflammation in three zones of the hepatic lobules. In comparison to the HFD group, the HFD + vinegar significantly reduced liver inflammation (*p* = 0.01) (Fig. 2).

### Effect of vinegar diet on HFD-induced alteration in serum TAC in the rats with NAFLD

HFD resulted in a decrease in serum TAC activity, compared to the SD group (*p* = 0.004). The serum TAC level was lower in the HFD group than in the HFD + vinegar group, although this difference was not statistically significant (Table 3).

### Effect of vinegar diet on HFD-induced alteration in hormone profile in the rats with NAFLD

**Fig. 3:**
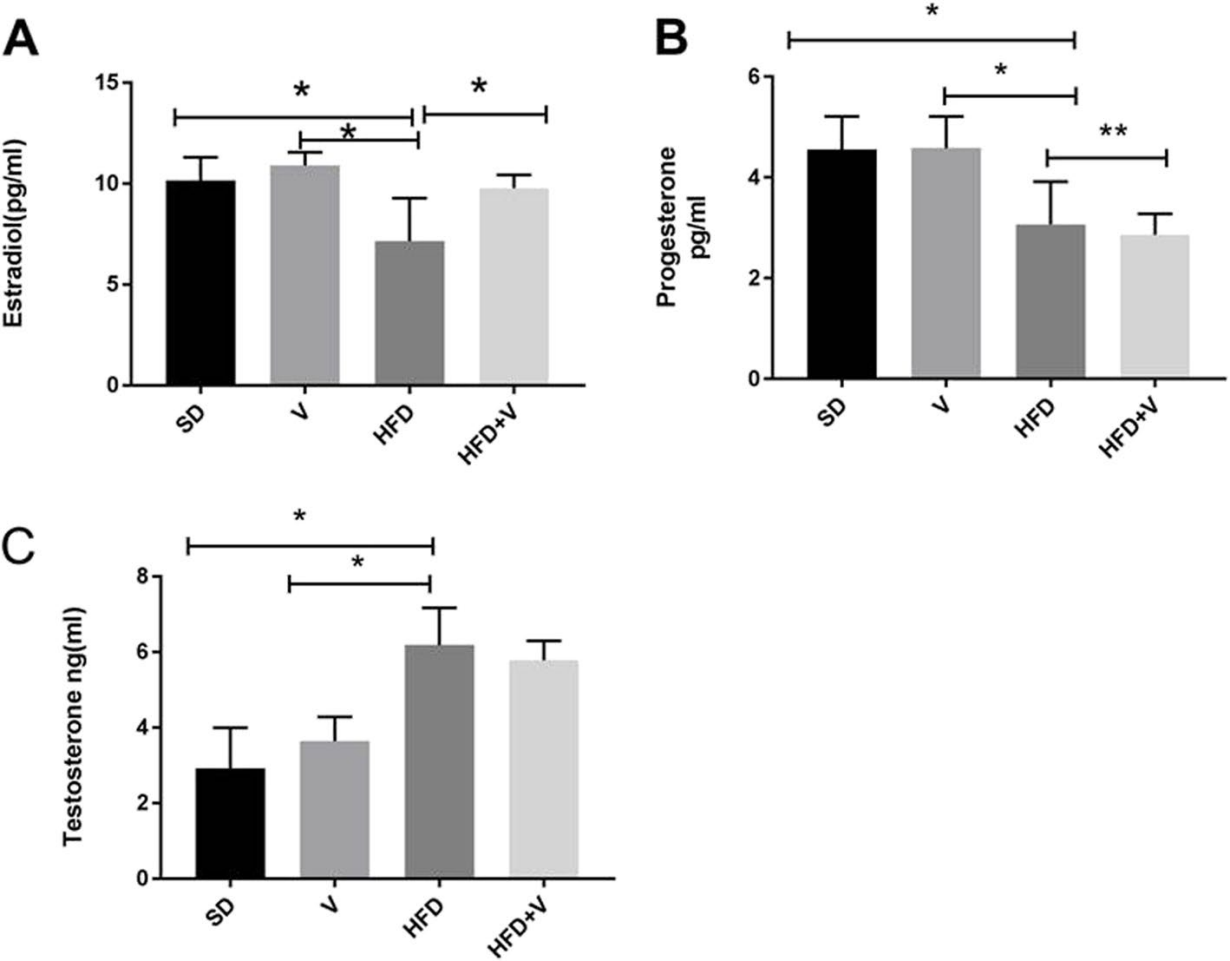
Serum concentration of estradiol, progesterone and testosterone in rats in different groups. Data are expressed as Mean ± SE. There are 7 rats in each group (*n* = 7). **A**: Estradiol data, *p = 0.001. **B**: progesterone data, *p = 0.001, **p = 0.01 and **C**: testosterone data *p = 0.001

The hormone profiles of the experimental groups were determined by serum testosterone, estradiol, and progesterone levels. The HFD group had lower estrogen and progesterone levels than the SD or SD + vinegar group (*p* = 0.001). (Fig. 3). Consumption of HFD + vinegar resulted in a significant rise in estradiol levels, compared to the HFD group (*p* = 0.001). Progesterone levels increased by a negligible amount in the HFD + vinegar group, compared to the HFD group. Serum testosterone levels were significantly greater in the HFD group than in the SD group (*p* = 0.001). In comparison to the HFD group, the HFD + vinegar combination was ineffective in lowering testosterone levels (Fig. 3).

### Effect of Vinegar diet on folliculogenesis in NAFLD animals

**Fig. 4:**
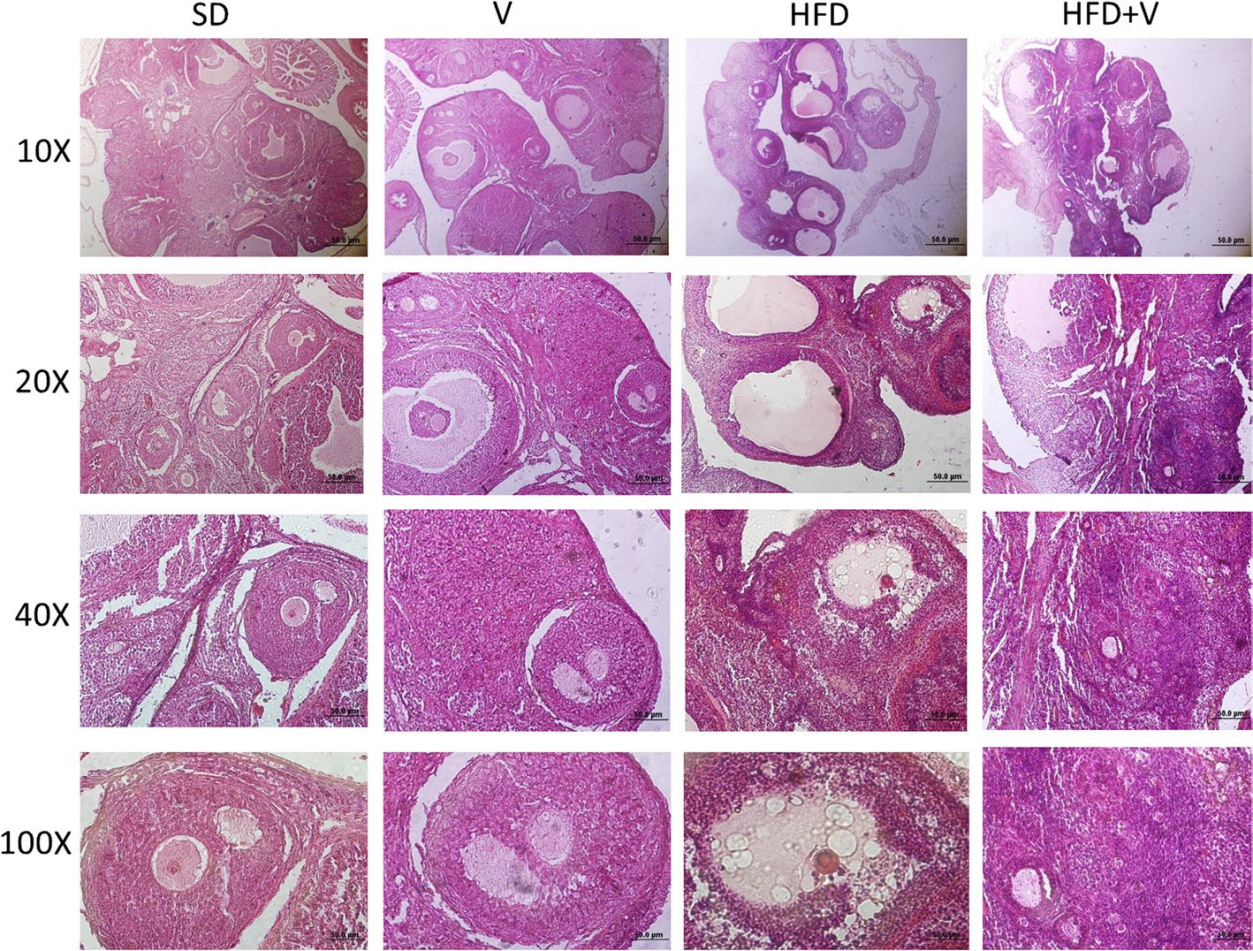
Photomicrograph of mouse ovary. Each column is showing a separate group. HFD has resulted an increase in degenerated follicles and cystic follicles. HFD + V has resulted in an increase in number of the primordial follicles. H & E staining

We studied H&E stained histology slides to determine the effect of the vinegar diet on folliculogenesis in rats with NAFLD. When compared to the SD group, the HFD dramatically reduced all types of follicles, including primordial, primary, secondary, pre-antral, and Graafian follicles, as well as corpus luteum (*p *< 0.05). Additionally, the HFD increased cystic and degenerative follicles, in contrast to the SD group over a 24-week period (*p* = 0.01). When compared with HFD, the HFD + vinegar increased the primordial and primary follicles (*p* = 0.001). In other words, the HFD + vinegar diet prevented the degeneration of primordial follicles, which are known as reserve follicles. There were no statistically significant variations in secondary, pre-antral, and Graafian follicles, as well as the corpus luteum, between the HFD + vinegar and HFD groups. No significant statisticaly differences were in cystic and degenerative follicles between HFD and HFD + vinegar groups (Table 4) (Fig. 4).

**Table 4:** Effect of vinegar on folliculogenesis in NAFLD rats

**Parameter**	**SD**	**HFD**	**SD + V**	**HFD + V**
**Primordial F**	7.0 ± 1.290	2.400 ± 0.509^a*^	6.666 ± 0.897^b**^	6.750 ± 0.453^b**^
**Primary F**	6.75 ± 0.487	1.750 ± 0478^a**^	6.555 ± 0.529^b**^	5.500 ± 626^b**^
**Secondary F**	9.50 ± 2.020	4.333 ± 0.614^a**^	8.777 ± 0.571^**b≠**^	6.0 ± 0.547
**Pre-antral F**	7.250 ± 1.108	2.833 ± 0.872^a**^	7.555 ± 0.444^b**^	5.375 ± 0.460
**Graaf F**	6.250 ± 0.629	1.250 ± 0.250^a**^	6.375 ± 0.323^b**^	2.750 ± 0.365^a**^
**Cystic F**	8.250 ± 1.030	16.571 ± 2.136^a*^	8.444 ± 0.626^b*^	11.750 ± 1.666
**Degenerative F**	8.750 ± 0.478	17.428 ± 1.674^a**^	7.444 ± 0.555^b**^	14.0 ± 1.592
**Corpus luteum**	18.50 ± 1.707	9.50 ± 0.957^a**^	18.0 ± 0.816^b**^	12.0 ± 0.816^**a≠**^

Data are expressed as Mean ± SE. There are 7 rats in each group (*n* = 7). a: Significant data in comparison to the SD, b: Significant data compared to the HFD group

^*^P = 0.01

^**^P = 0.001

≠ *P* = 0.04

### Effect of vinegar diet on proliferation indexes of granulosa cells in the rats with NAFLD

**Fig. 5:**
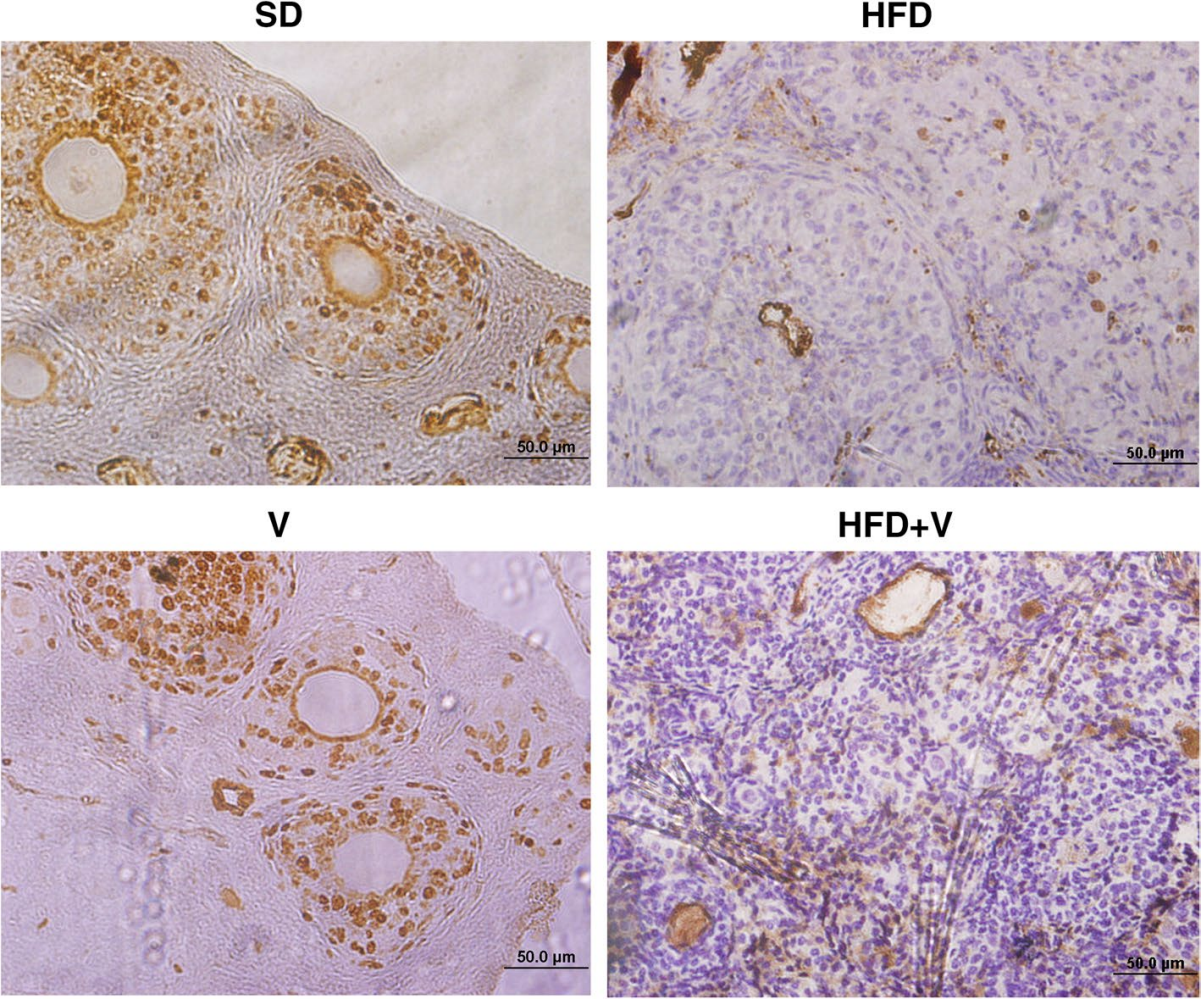
Ki-67 immunostaining in rat ovary tissue. Brown cells are KI-67 positive cells. a weak immunostaining reaction in granulosa cells is noted in HFD group. HFD + vinegar has not significant effect in granulosa cell proliferation index in compare with HFD group. 200x

Immunohistochemistry was used to determine the proliferation indexes of granulosa cells, using an anti-rat ki-67 marker. The index of granulosa proliferation was not significantly different between the groups in primordial follicles. However, when compared to the SD group, HFD lowered the granulosa proliferation index in primary, secondary, pre-antral, Graafian follicles, and corpus luteum after 24 weeks (*p* = 0.001). Vinegar + HFD had no statistical significant effect on the proliferation indexes of granulosa cells, in a variety of follicle types (Table 5) (Fig. 5).

**Table 5:** Granulosa cell proliferation index in NAFLD rat models treated with vinegar

**Parameter**	**SD**	**HFD**	**SD + V**	**HFD + V**
**Primordial F**	37.75 ± 1.314	33.25 ± 1.25	36.5 ± 3.227	33.5 ± 1.443
**Primary F**	50 ± 3.135	16 ± 2.160^a*^	48 ± 2.581^b*^	24.25 ± 0.853^a*^
**Secondary F**	61.25 ± 2.688	31.25 ± 1.732^a*^	55.25 ± 1.701^b*^	38.50 ± 3.013^a*^
**Pre-antral F**	57.50 ± 2.66	33.0 ± 1.914^a*^	63 ± 2.081^b*^	36.5 ± 1.554^a*^
**Graaf F**	46 ± 1.779	24 ± 1.683^a*^	32.75 ± 0.208^b*^	36.66 ± 0.134^a*^
**Corpus luteum**	31.75 ± 2.495	28.25 ± 0.853	33 ± 1.080^b**^	32.75 ± 1.60

Data are expressed as Mean ± SE. There are 7 rats in each group (*n* = 7). a: Significant data in comparison to the SD, b: Significant data compared to the HFD group

^*^P = 0.001

^**^P = 0.04

### Vinegar affected the expression levels of ovarian kisspeptin in the rats with NAFLD

**Fig. 6:**
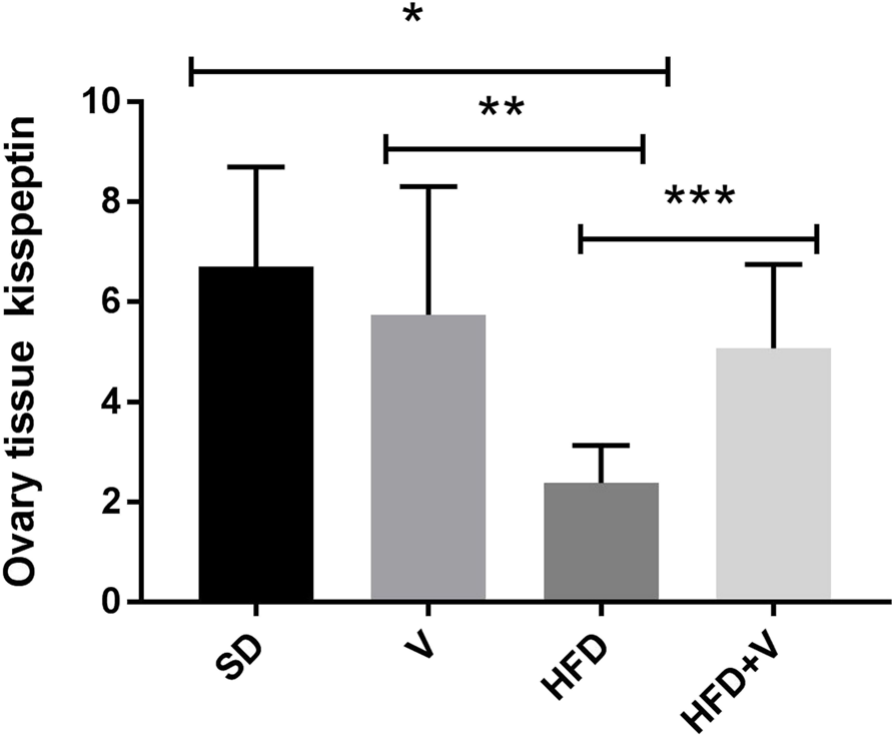
Expression of Kisspeptin in ovary tissue in rats in different groups. Data are expressed as Mean ± SE. There are 7 rats in each group (*n* = 7). *P = 0.01, **P = 0.03 and ***P = 0.04

To elucidate the molecular mechanisms behind the diets' effect, we assessed the kisspeptin expression in ovarian tissue at the end of 24 weeks. Our real-time PCR data indicated that the HFD significantly decreased the kisspeptin expression in ovarian tissue, compared to the SD group (*p* = 0.01). We found no difference in ovarian kisspeptin expression between the SD and SD + vinegar diets. HFD + vinegar significantly enhanced the kisspeptin expression, compared to the HFD group (*p* = 0.04) (Fig. 6).

## Discussion

The results of this study showed that consumption of 60% kcal HFD during 24 weeks in female rats induced NAFLD, demonstrated by steatosis and liver histology, hyperlipidemia, hyperglycemia, insulin resistance, and abnormal liver enzymes. NAFLD also resulted in impaired folliculogenesis, disruption of sex hormones, and reduction of kisspeptin expression in the ovary. Generally, obesity is associated with impaired fertility, which often phenotypically presents as higher incidences of poor oocyte quality, irregular menstrual cycles, anovulation, and increased miscarriage rates; suggestive of derangements in ovarian folliculogenesis and steroidogenesis [23–25]. Vinegar consumption in the HFD group alleviated hyperlipidemia, hyperglycemia, and decreased liver enzymes in this study. We conclude that vinegar has anti-obesity properties by lowering the Lee index. In fact, acetic acid was thought to be the key element in vinegar that influenced body fat loss and weight growth. Furthermore, consuming apple vinegar induces fat dispersion, which facilitates lipase action on adipose tissue, suppresses body fat accumulation, inhibits cytoplasmic lipid accumulation, regulates adipogenesis, and therefore eliminates body fat [26, 27].

Similarly, Kondo et al., found that drinking 15 and 30 ml of apple vinegar daily for 12 weeks reduced lipid profile and obesity in a clinical investigation of Japanese patients [13].

Apple cider vinegar can treat insulin resistance and hyperglycemia [28]. Anti-glycemic properties of apple vinegar in this experiment may also be related to the vinegar’s acetic acid levels. Acetic acid through slowing down gastric emptying, suppressing disaccharide activity in the small intestine, limiting the complete digestion of starch molecules, and also enhancing glucose uptake by physical performance, has anti-glycemic effects [26, 27].

There is a link between hepatic steatosis and insulin resistance, and numerous rat models have been used to show that lower hepatic TG pools are linked to better insulin sensitivity [29, 30]. HFD-produced mild steatosis was confirmed by biochemical and histological analyses in this study. While the vinegar diet lowered steatosis from 59.85 to 32.65 percent, which is considered mild, confirming that apple vinegar reduces steatosis from moderate to mild.

Our data indicated a four-fold increase in serum TAC in the vinegar + HFD group. Antioxidants have been proposed as potential therapeutic agents for NAFLD and related illnesses [31].

Flavonoids are a class of naturally occurring antioxidants found in a variety of fruits, plants, and other plants. The hydroxyl groups in flavonoids' phenolic structure are particularly important for their antioxidant properties. Vinegar is rich in flavonoids [31]. It is possible that in this study vinegar through its antioxidant effect could ameliorate NAFLD signs.

Our data demonstrated that HFD-caused NAFLD adversely affected all types of follicles, including primordial, primary, secondary, pre-antral, Graafian, and corpus luteum. HFD significantly raised the number of degenerative and cystic follicles. Primordial follicles are the most fundamental component and make up the ovarian reserves in mammals. They are produced just in the prenatal period and their growth is independent of gonadotropins. Only a few primordial follicles are typically triggered at any given time, with the majority of them remaining in a dormant state under normal conditions [32]. In line with our work, HFD reduces primordial follicles in rats [33] and mice [34]. Similar to our study, curcumin as a main natural polyphenol protects the primordial follicles from overactivation [32]. Follicle loss may be related to some factors such as hormonal abnormalities, ROS elevation, reduction of enzymatic and non-enzymatic antioxidants, the elevation of apoptotic markers, and proliferation index reduction. All of the above-mentioned variables may lead to reproductive issues in women. In this study, both estrogen and progesterone were decreased in the HFD-induced NAFLD group. Estrogen is a critical hormone in both the development and normal physiology of the female reproductive tract.

The granulosa and interna theca cells synthesize estradiol in pre-ovulatory follicles, following stimulation by Follicle Stimulating hormone (FSH). Estradiol is important for LH receptor expression, the development of antrum and gap junctions, and also follicular atresia prevention. It is also important for the proliferative phase in the uterus [35].

Our findings about the decline in the number of all types of follicles in the HFD group were consistent with the previous results of research in diabetics [36, 37].

Our study demonstrated corpus luteum as a measure of ovulation was reduced in an animal model of NAFLD. Corpus luteum secrets progesterone. Therefore, the less corpus luteum development, the less progesterone secretion will be. Our findings verified the reduced serum levels of progesterone in an animal model of NAFLD. Nteeba et al*.* have shown that growing obesity modifies the expression of inflammatory and steroidogenesis signaling pathway members in a manner that could modify the optimal ovarian function in mice [23]. Likely, Jacob S. Roberts et al. have shown that HFD causes Polycystic ovarian syndrome (PCOS) in female rats and lowers all types of follicles and corpus luteum [6]. Additionally, Mona A. Hussain reported a decrease in corpus luteum following obesity and HFD, in a rat model [38]. Our results suggested supplements of apple vinegar and HFD enhanced the numbers of primordial and primary follicles, without having a significant effect on the other types of follicles and corpus luteum. The exact mechanisms are unknown. Little is known regarding the influence of vinegar on folliculogenesis in NAFLD models.

Insulin resistance deregulates the genes, enzymes, and proteins necessary for the steroidogenic machinery, ultimately impairing follicle formation and ovulation [39], and our findings in NAFLD animals on an HFD validated that steroidogenesis was altered. We detected a decrease in insulin resistance in animals fed with a vinegar-containing HFD. This may be because of weight loss, as Shah et al. have shown in clincal research that branched-chain amino acid levels are linked to improved insulin resistance following weight loss [40]. In HFD-induced NAFLD, we detected an increase in serum testosterone levels in conjunction with obesity. Previous research has suggested that hyperandrogenism, a distinctive characteristic of PCOS, may contribute independently to the development of NAFLD in women with PCOS [17]. Possibly, an earlier study discovered that obese women with PCOS had considerably greater total serum testosterone levels than non-obese women with PCOS [41]. However, Tsai et al., demonstrated that in the PCOS group, blood testosterone levels were not substantially different between obese and non-obese subjects [42].

In this study disruption of folliculogenesis and steroidogenesis in NAFLD animals may be a result of ovarian kisspeptin changes, which our analysis corroborated. We found a decrease in ovarian kisspeptin expression in NAFLD rats. Kisspeptin is a novel hypothalamic peptide that enhances endogenous GnRH release [7]. Both kisspeptin and its receptor are localized in the stroma of the GnRH neurons in the preoptic area and also in axon terminals in median eminence [43]. Additionally, the presence of kisspeptin and its roles have been shown in peripheral organs such as the ovary and testis [43–45]. Kisspeptin has been found in the ovary both in rats and in humans [43, 44]. Administration of kisspeptin in aging rats resulted in an increase in corpus luteum and folliculogenesis [43, 44].

Female rats exposed to an HFD had a similar long-term effect on reproductive function, including earlier onset of puberty and disruption of estrous cyclicity during puberty and maturity [10]. Kisspeptin has been implicated in the control of ovarian steroidogenesis and folliculogenesis [46].

Kisspeptin expression in the ovary was decreased in the NAFLD groups in our study. Kisspeptin also is important in regulating metabolic processes [44]. Both of these are done directly via the effect on kisspeptin receptors in the ovaries, brain, pancreas, or brown adipose tissue. Kisspeptin also can regulate metabolic processes indirectly via gonadal hormones [44, 47–49]. Previously, it was established that elevated plasma TGs are related to decreased kisspeptin levels in obese rats [47]. In obese people, a high body mass index (BMI) is associated with a high serum TG level [48]. Indeed, kisspeptin and BMI have a negative association. Additionally, obese female individuals have lower kisspeptin concentrations than non-obese female participants [49]. Reduced kisspeptin concentrations in obese people are almost certainly correlated with problems associated with alterations in the sex hormones that regulate obesity and puberty [49].

Panidis et al. discovered that women with PCOS had lower serum kisspeptin levels than controls [50]. Our data most likely indicated a higher prevalence of cystic follicles in the NAFLD group. Kisspeptin appears to be involved in the regulation of glucose and/or fat metabolism, which may be relevant in the context of obesity or type 2 diabetes. In this context, based on a clinical investigation, serum insulin and glucagon concentrations, as well as HOMA-IR, are adversely linked to kisspeptin [49]. Our study revealed that vinegar + HFD increased the expression of kisspeptin, in comparison to the HFD. Similarly, Peng et al. demonstrated that the flavonoids in apple vinegar can enhance PI3K / AKT signaling by improving fat and sugar metabolism and increasing kisspeptin expression in the ovary [51]. Increasing primordial and primary follicles, estradiol level, and kisspeptin expression in the ovary following vinegar + HFD may be due to flavonoid content and antioxidant properties of the vinegar. However, the exact mechanisms are unknown. In this study, we did not evaluate the kisspeptin receptor expression. However, we showed anti-glycemic, anti-lipidemic, and anti-obesity effects of vinegar + HFD. We also showed an increase in estradiol levels in the vinegar + HFD group. All these alterations indicate the indirect effect of vinegar on ovarian kisspeptin expression. The exact mechanisms of vinegar and ovary kisspeptin are unknown and need further investigation.

Our study limitation in this study was using rats as the NAFLD model. Numerous animal models have been utilized to research obesity and obesity-related ovarian dysfunction. However, a lot of these models use mouse strains that have undergone genetic modification, are fed high-fat diets, and may not accurately reflect the pathophysiology of human obesity [52]. Additionally, rodents have a limited capacity to monitor longitudinal changes in biochemical parameters over time. Although obesity in non-human primates closely resembles the symptoms of the disease manifestation in humans, their price is high. On the other hand, they have a considerable delay before they reach adulthood. Moreover, due to the higher risk of zoonotic illness in these animals, huge populations cannot access them [52]. Additionally, no single animal model can reproduce a complete picture of the mechanisms responsible for the appearance, variations, progression, and outcome of human disease [52, 53]. Vinegar is a safe, cheap and available solution. Therefore, the study of the effect of vinegar in NAFLD women on folliculogenesis, sex hormones, and the involved mechanisms is recommended for further studies.

## Conclusion

The vinegar diet in a rat model of NAFLD raises estradiol, primordial, and small primary follicles, and increases ovarian kisspeptin expression indirectly. Insulin resistance and obesity were improved by apple vinegar, and anti-glycemic and anti-lipidemic effects were also determined. The supplementation of apple vinegar in NAFLD might be useful. However, it requires further investigation.

## Data Availability

All data during this study are included in this published article. Availability of data and materials The datasets used and/or analysed during the current study available from the corresponding author on reasonable request.

## Abbreviations

HFD: High –fat- diet
SD: Standard diet
NAFLD: Nonalcoholic fatty liver disease
HOMA-IR: Homeostatic model assessment for insulin resistance
GnRH: Gonadotrophin-releasing hormone
LH: Luteinizing hormone
IVF: In vitro fertilization
ALT: Alanine aminotransferase
AST: Aspartate aminotransferase
TG: Triglyceride
HDL: High-density lipoprotein
TAC: Total antioxidant capacity
H & E: Hematoxylin and eosin
KS: Kolmogorov–Smirnov
